# A meta-analysis of elevated O_3_ effects on herbaceous plants antioxidant oxidase activity

**DOI:** 10.1371/journal.pone.0305688

**Published:** 2024-06-25

**Authors:** Yi Zhao, Bing Guo, Zhouli Liu, Xiaohan Wang, Guangmin Xiao, Roland Bol

**Affiliations:** 1 School of Chemistry and Environmental Engineering, Liaoning University of Technology, Jinzhou, Liaoning, China; 2 Institute of Bio- and Geosciences, Agrosphere (IBG-3), Forschungszentrum Jülich GmbH, Jülich, Germany; 3 College of Life Science and Engineering, Shenyang University, Shenyang, China; 4 Key Laboratory of Black Soil Evolution and Ecological Effect, Ministry of Natural Resources, Shenyang, China; 5 Institute of Agricultural Resources and Environment, Hebei Academy of Agriculture and Forestry Sci-ence/Hebei Fertilizer Technology Innovation Center, Shijiazhuang, China; 6 School of Natural Sciences, Environment Centre Wales, Bangor University, Bangor, United Kingdom; Nuclear Science and Technology Research Institute, ISLAMIC REPUBLIC OF IRAN

## Abstract

Increases in near-surface ozone (O_3_) concentrations is a global environmental problem. High-concentration O_3_ induces stress in plants, which can lead to visible damage to plants, reduced photosynthesis, accelerated aging, inhibited growth, and can even plant death. However, its impact has not been comprehensively evaluated because of the response differences between individual plant species, environmental O_3_ concentration, and duration of O_3_ stress in plants. We used a meta-analysis approach based on 31 studies 343 observations) to examine the effects of elevated O_3_ on malondialdehyde (MDA), superoxide dismutase (SOD), and peroxidase (POD) activities in herbaceous plants. Globally, important as they constitute the majority of the world’s food crops. We partitioned the variation in effect size found in the meta-analysis according to the presence of plant species (ornamental herb, rice, and wheat), O_3_ concentration, and duration of O_3_ stress in plants. Our results showed that the effects of elevated O_3_ on plant membrane lipid peroxidation depending on plant species, O_3_ concentration, and duration of O_3_ stress in plants. The wheat SOD and POD activity was significantly lower compared to the herbs and rice (*P<0*.*01*). The SOD activity of all herbaceous plants increased by 34.6%, 10.5%, and 26.3% for exposure times to elevated O_3_ environments of 1–12, 13–30, and 31–60 days, respectively. When the exposure time was more than 60 days, SOD activity did not increase but significantly decreased by 12.1%. However, the POD activity of herbaceous plants increased by 30.4%, 57.3%, 21.9% and 5.81%, respectively, when exposure time of herbaceous plants in elevated O_3_ environment was 1–12, 13–30, 31–60 and more than 60 days. Our meta-analysis revealed that (1) rice is more resistant to elevated O_3_ than wheat and ornamental herbs likely because of the higher activity of antioxidant components (e.g., POD) in the symplasts, (2) exposure to elevated O_3_ concentrations for >60 days, may result in antioxidant SOD lose its regulatory ability, and the antioxidant component POD in the symplast is mainly used to resist O_3_ damage, and (3) the important factors affected the activity of SOD and POD in plants were not consistent: the duration of O_3_ stress in plants was more important than plant species and O_3_ concentration for SOD activity. However, for POD activity, plant species was the most important factor.

## 1. Introduction

Surface ozone (O_3_) is one of the main air pollutants and a powerful greenhouse gas, and its concentrations are increasing globally and regionally [1]. The near-surface O_3_ concentration has now reached the global average of approximately 50 nmol·mol^-1^ [2], which exceeds the appropriate concentration (40 nmol·mol^-1^) for plant growth [3], increasing at an annual rate of 0.5%–2% over the past decades [4]. In the past two decades, elevated O_3_ levels in many countries have been sufficient to cause damage to plants, especially in some areas with high O_3_ concentrations, causing huge economic losses [5–10]. The toxic effects of O_3_ on plants are mainly due to its strong oxidation ability, which is a concern. Elevated O_3_ is taken by plants through stomata, causing visible damage to leaf [11], reduces photosynthesis [12,13], accelerates plant aging [14], inhibits plant growth [15,16], and can even lead to plant death. When plants are subjected to O_3_ stress, they can adapt to (or slow down) the stress of elevated O_3_ levels by adjusting their physiological and biochemical metabolic pathways, such as by reducing stomatal conductance, increasing antioxidant activity, increasing the synthesis of antioxidant substances, and enhancing the expression of antioxidant genes, which improves their self-protection and repair ability [16,17].

Herbaceous plants are an important part of ecosystems. They exist partly as crops and partially as ornamental plants. They provide the necessary food for people and storage as well as play an important role in regulating the ecological environment. Regarding ecological effects, ornamental plants have the functions of reducing dust, reducing noise, conserving water and soil, absorbing toxic gases, and reducing air pollution [18]. Therefore, some studies consider that ornamental plants can be used as indicators of O_3_ pollution [19,20]. Researchers are more concerned with the yield and food security of herbaceous crops in elevated O_3_ environments [7,10]. Different plants have different adaptation and defense capabilities to elevated O_3_, and the degree of influence of O_3_ on plants and the ability of plants to adapt to O_3_ also depend on the sensitivity of plants to O_3_ [21]. Grulke and Heath (2020) reported that herbaceous crops are more sensitive to O_3_ than deciduous woody plants, conifers, and grasses [11]. However, previous studies have mainly focused on individuals of different plant genera, with no systematic and comprehensive comparisons. Some results of previous independent studies have shown differences in the response of different species of herbaceous plants to elevated O_3_ [19], but it is necessary to assess the overall effect on a global scale.

As the most important defense system in plants, the study of antioxidant systems will inevitably help reveal the response mechanisms of plants to high O_3_ concentrations. We searched for the keywords O_3_, plant, and stress in the Web of Science (WOS) and imported the retrieved literature into the VOSviewer bibliometric software (S1 Fig in S1 File). The results showed that previous studies on the effects of O_3_ on plants have primarily focused on plant growth, yield, sensitivity, and oxidative stress [5,6,21]. More researches have focused on one-species photosynthetic indicators (such as chlorophyll and stomatal conductance). The investigation of the effects of elevated O_3_ on vegetation antioxidant systems is more complex than the impacts of yield and photosynthesis, and the available information is limited and includes controversial results. In this state, meta-analysis is an important statistical analysis method that integrates the results of previous independent studies to assess the overall effect and arrive at more reliable results globally. At present, no meta-analysis has been carried out on the effects of elevated O_3_ on the antioxidant systems of different herbaceous plants, limiting our exploring an effective strategy to inhibit or alleviate the harm caused by elevated O_3_ to plants on global terrestrial ecosystems. Therefore, it is necessary to integrate the results of the previous experimental researches to comprehensively assess the effects of elevated O_3_ on various types of herbaceous plants, which will provide scientific basis for the study of plant adaptation mechanism to O_3_ stress under global change.

Malondialdehyde (MDA) is a product of membrane lipid peroxidation when plant organs age or are under stress, and its content reflects the degree of membrane lipid peroxidation in plants [22]. Superoxide dismutase (SOD) and peroxidase (POD) are the most important protective enzymes in the plant antioxidant system; they can effectively remove reactive oxygen species (ROS) produced during metabolism and prevent membrane lipid peroxidation and damage caused by ROS [13,23]. To comprehensively analyze the effect of elevated O_3_ on herbaceous plants and the response of the antioxidant indices of herbaceous plants to O_3_, we performed a meta‐analysis of the available information (31 studies, 343observations) to examine the effects of elevated O_3_ levels on MDA content and SOD and POD activities in herbaceous plants. The main objective of this meta‐analysis is to address the following three questions: (1) Whether different plant species, different O_3_ concentration and different stress time have significant effects on the damage of plant membrane lipid in a high concentration O_3_ environment; (2) whether the antioxidant enzyme activity of ornamental herbs and herbaceous crops (rice and wheat) is similar in response to elevated O_3_; (3) Plant species, O_3_ concentration, and stress time which one is the most important factor affecting the response of plant SOD and POD enzyme activity to elevated O_3_?

## 2. Material and methods

### 2.1 Data source

**Fig 1:**
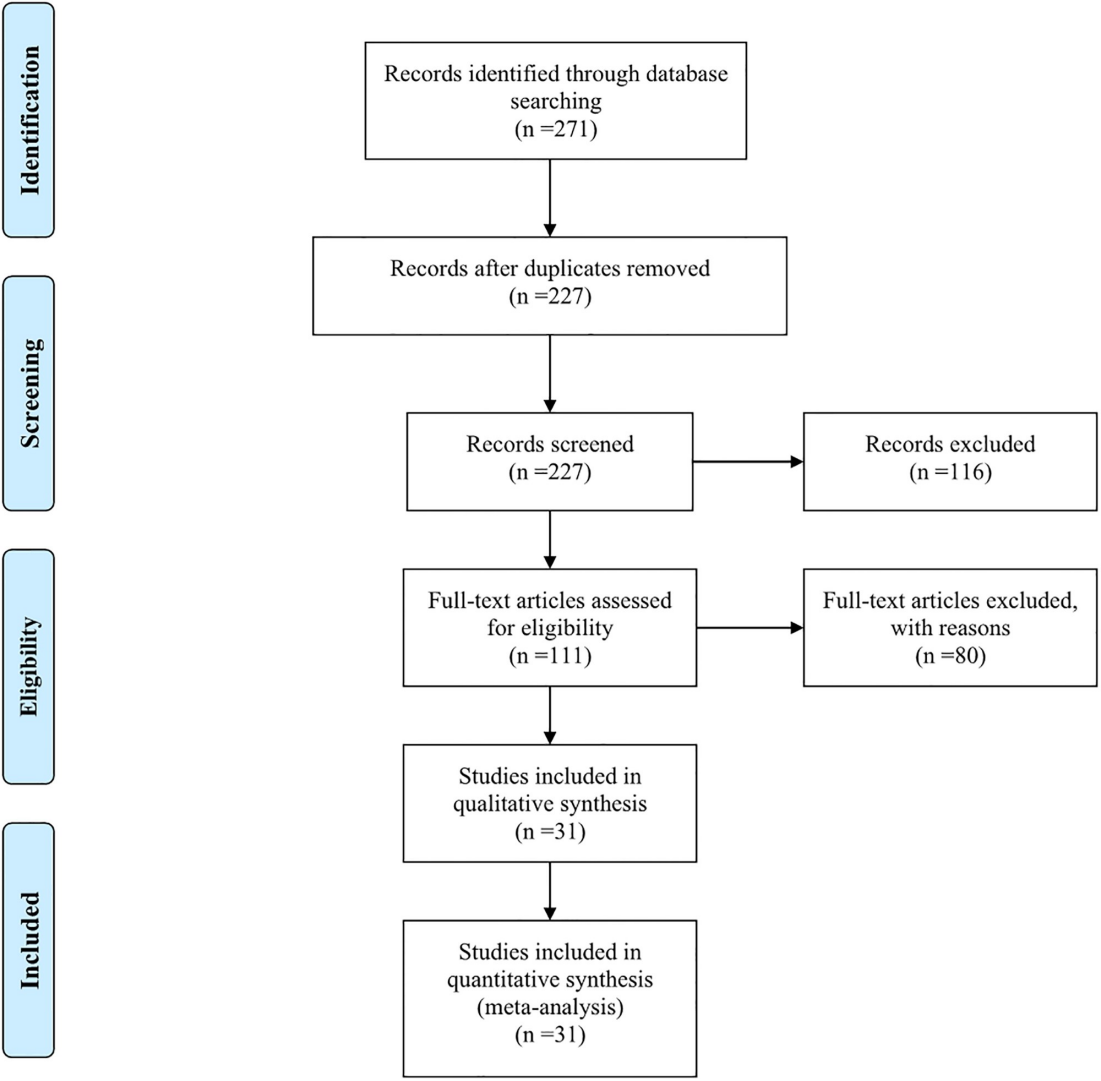
The PRISMA flowchart of database query and study selection process.

A survey of peer-reviewed papers was published by the WOS and China National Knowledge Infrastructure (CNKI). All peer-reviewed literature published on this topic between 1990 and 2024 (the most recent search date for the literature is February 8, 2024) were obtained using the keywords: “herbaceous plants,” “ornamental herb,” “grass,” “rice,” “wheat,” “ozone or O_3_ or elevated O_3_ or O_3_ stress or environmental O_3_ concentration” and “impact or respond or affect or alter or effect.” The relevant research reports were 121 publications in the WOS and 150 publications in CNKI. And then160 researches which were repetitive studies and did not conform to the subject of this research were eliminated, by read their abstracts and material methods (Fig 1). After examining the relevance of duplications in the WOS and CNKI, 111 publications (46 publications in the WOS and 65 publications in the CNKI) were identified for further screening (Fig 1).

**Fig 2:**
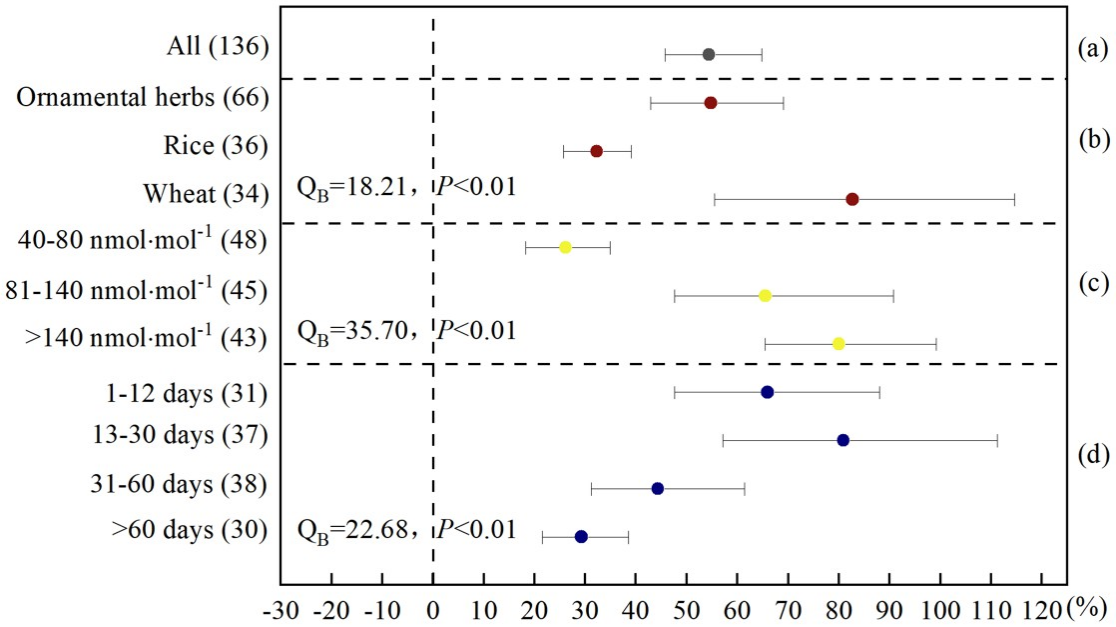
Meta-analysis of the effects of elevated O_3_ on MDA content of herbaceous plants. (a) categorized into (b) plant species, (c) O_3_ concentration, and (d) experiment duration. MDA content responses were expressed as the relative increase (%) compared to the control (CK), with 95% CIs represented by the error bars. The numbers of paired observations are shown in parentheses. Between-group heterogeneity (Q_B_) and between-group probability (*P*) was used to describe the statistical differences in MDA content responses between the different levels of the categorized factors.

To include a research report in this meta-analysis, we examined if it met the following criteria (Fig 2): (1) plant species were rice or wheat or ornamental herbs; (2) plants were exposed to elevated O_3_ at least for 1 day; (3) O_3_ concentration in the control treatment (CK) of the experiment was less than 40 nmol·mol^-1^; (4) environmental O_3_ concentrations were higher (40 nmol·mol^-1^) in treatments during exposure; (5) the research indices included MDA content, SOD and POD activities, and (6) the means, standard deviation (SD) or standard error (SE), and replicate numbers (n) were recorded clearly and could be obtained. The final database consisted of 31 research reports published, including 10 articles indexed by the WOS and 21 studies indexed by the CNKI. All data were obtained from tables or extracted from figures using the graph digitizing software (GetData Graph Digitizer V2.25; http://getdata-graph-digitizer.com/). A total of 343 sets of observations were included in the meta-analysis.

To distinguish the sources of variation in the responses of MDA content and SOD and POD activities to elevated O_3_, the paired measurements were further subdivided into subgroups according to the categorical variables listed in Table 1.

**Table 1:** Classification of categorical variables used as explanatory factors.

Categorical variable	Level 1	Level 2	Level 3	Level 4
**Plant species**	ornamental herbs	rice	wheat	-
**O_3_ concentration (nmol·mol^-1^)**	40–80	81–140	> 140	-
**Experiment duration (days)**	1–12	13–30	31–60	> 60

### 2.2 Meta-analyses

The meta‐analysis method was as that described in Hedges et al. (1999) [24].

The effect size is an index that reflects the magnitude of the elevated O_3_ effect in comparison with the CK treatment [25]. In this study, the effect size of each observation (taken to be the comparison between elevated O_3_ and CK) for MDA content, SOD and POD activities was calculated as the natural log of the response ratio (RR), which was used as the metric for analysis [24,26], as shown in Eq (1):

$$\text{ln}\;RR=\text{ln}\;\frac{X_{t}}{X_{c}}$$ (1)

where X_t_ and Xc are the mean values of groups exposed to elevated O_3_ treatment and CK treatment, respectively.

The variance (V) of R was calculated as shown in Eq (2):

$$V=\frac{SD_{t}^{2}}{N_{t}X_{t}^{2}}+\frac{SD_{c}^{2}}{N_{C}X_{C}^{2}}$$ (2)

where SD_t_ and SD_c_ are the standard deviations for the elevated O_3_ treatment and the CK treatment, and N_t_ and N_c_ are the sample sizes for the elevated O_3_ treatment and the CK treatment, respectively. The random effect model was used to calculate the weighed response ratio (RR_++_), and bootstrapping (9999 iterations) was used to assess 95% confidence intervals (95% CI) for the average effect size and bias correction for each categorical variable [5,27]. The relative change in the effects of elevated O_3_ is reported as a percentage [28], as shown in Eq (3):

$$change\left(\%\right)=\left(e^{RR_{++}}-1\right)\times 100\%$$ (3)

Positive percentage changes indicated an increase in the herbaceous plant variables in response to the elevated O_3_ treatment relative CK, whereas negative values indicated a decrease. Significant responses (p < 0.05) were considered if the 95% CIs did not overlap with zero [28,29].

To examine whether differences between individual plant species, environmental O_3_ concentration, and duration of O_3_ stress in plants alter the effects of O_3_ on plant growth, the categorical analysis was proceeded across all data by dividing the Q_T_ into the within-group heterogeneity (Q_W_) and between-group heterogeneity (Q_B_). The analyses were performed with MetaWin 2.1 statistical software (Sinauer Associates Inc.) [26]. The Q_T_ of each variable was tested against a χ^2^-distribution with degrees (n-1) of freedom. If the test result indicated no heterogeneity (*P>0*.*05*), chose the fixed effect model; if *P<0*.*05* chose the random effect model [30]. In this study, the Q_T_ tested results of MDA, SOD and POD were *P<0*.*05* (Table 2), we chose a random model. It was accepted that the mean effect sizes of the categories were significantly different between the levels of the factors if the *P* values of the Q_B_ were less than the 0.05 level (*P<0*.*05*) [31,32].

**Table 2:** The total heterogeneity (Q_T_) and Rosenthal’s fail-safe numbers in this study.

	Observation number (k)	Q_T_	Rosenthal’s fail-safenumbers ^a^
**Malondialdehyde (MDA)**	136	173.6*	12026.9
**Superoxide dismutase (SOD)**	111	209.6**	1318.1
**Peroxidase (POD)**	96	150.2**	881.3

*P*-values < 0.05 are considered significant.

* *P* < 0.05;

** *P* < 0.01.

^a^ No publication bias if the Rosenthal’s fail-safe numbers are greater than (5k + 10).

The possibility of publication bias of MDA, SOD and POD were evaluated with Rosenthal’s fail-safe number at α = 0.05 (Table 2) [30,33]. No publication bias was existed if Rosenthal’s fail-safe number was more than 5k + 10, where k is the observation number [30,33].

Random forest algorithm is suitable to handle large numbers of variables without assuming their causalities [34]. In this study R programming language version 3.5.1, the random forest algorithm, was used evaluate the relative importance (%IncMSE) of factors influencing MDA content, SOC and POC activity.

## 3. Results and discussion

### 3.1 Effects of elevated O_3_ on oxidative stress of herbaceous plants

Considering the entire dataset of the overall effects of O_3_ on MDA were positive, elevated O_3_ increased the MDA content of herbaceous plants by 54.4% (n = 136) (Fig 2a). When the data were subdivided by plant species, the elevated O_3_ increased the MDA content of ornamental herbs, rice and wheat by 54.8%, 32.3% and 82.7%, respectively (Fig 2b); When the data were considered to distinguish by environmental O_3_ concentration, the MDA content was increased by 26.1%, 65.5%, and 80%, respectively, in the O_3_ environment of 40–80, 81–140, and >140 nmol·mol^-1^ (Fig 2c); When the data were subdivided by duration of O_3_ stress in plants, the plants were subjected to elevated O_3_ stress for 1–12, 13–30, 31–60, and more than 60 days, with MDA content increases of 65.9%, 80.9%, 44.3%, and 29.3%, respectively (Fig 2d).

The selective permeability of the protoplasmic membranes of plant cells is one of the most important functions of plants, and membrane peroxidation is considered an important characteristic of plant oxidative damage under O_3_ stress [35]. MDA is one of the main products of membrane lipid peroxidation when plants are subjected to elevated O_3_ stress, and its content reflects the degree of membrane lipid peroxidation: the higher the MDA content, the more damaged the plant [36]. Our global synthesis showed that when the concentration of O_3_ in the environment exceeds the natural state (40 nmol·mol^-1^), it will cause membrane lipid peroxidation damage to herbs. The effects of elevated O_3_ on plant membrane lipid peroxidation depending on plant species, O_3_ concentration, and duration of O_3_ stress in plants.

In this study, the response of MDA content of different plant species to elevated O_3_ concentration was significantly different. The order of MDA content increased was wheat > ornamental herbs > rice, i.e. elevated O_3_ was the most damage to wheat and the least damage to rice. Compared to wheat and ornamental herbs, rice causes the least O_3_ damage, which may be due to two reasons. (1) The antioxidant system of rice may be stronger than that of wheat and ornamental plants. When O_3_ enters rice leaves through the stomata, a rapid antioxidant enzyme reaction may occur, and the damage caused by O_3_ to rice may be reduced over time. (2) Elevated O_3_ and other environmental factors in which plants are located may interact and affect the response of plants to stress [37–39]. Compared with wheat and ornamental herbs, the growth environment of rice promotes a richer water supply, which may mitigate elevated O_3_ oxidation damage.

The effects of different O_3_ concentrations on the MDA content of herbaceous plants were significantly different (Fig 2c). The MDA content in herbaceous plants increased with an increase O_3_ concentrations in their environment, which is consistent with other studies on the effect of O_3_ on membrane lipid peroxidation in trees [16,40].

The decreasing magnitudes occurred for responses of MDA content to duration of O_3_ stress in plants from short time to long time (Fig 2d). When the duration of O_3_ stress in plants was more than 60 days, the increase in MDA content significantly decreased (*P<0*.*01*), only increasing by 29.3% (21.6%–38.6%). Two possible reasons are: (1) when herbaceous plants are exposed to O_3_ for too long (more than 60 days), they are irreversibly damaged, causing their cells to die, resulting in lower MDA levels that can be measured, or (2) the experiment with more than 60 days of stress is likely to adopt a low treatment concentration of 40–80 nmol·mol^-1^. This concentration is close to the O_3_ concentration in areas with serious O_3_ pollution [41–43], and plants are not only passive recipients of external stress but also can quickly perceive and actively adapt to changes in environmental conditions [44]. When herbs are in such an environment for a long time, they may gain stronger resistance, thus reducing the damage caused by O_3_ to membrane lipid peroxidation and decreasing MDA content.

### 3.2 Effects of elevated O_3_ on the activities of antioxidative enzymes in leaves of herbaceous plants

**Fig 3:**
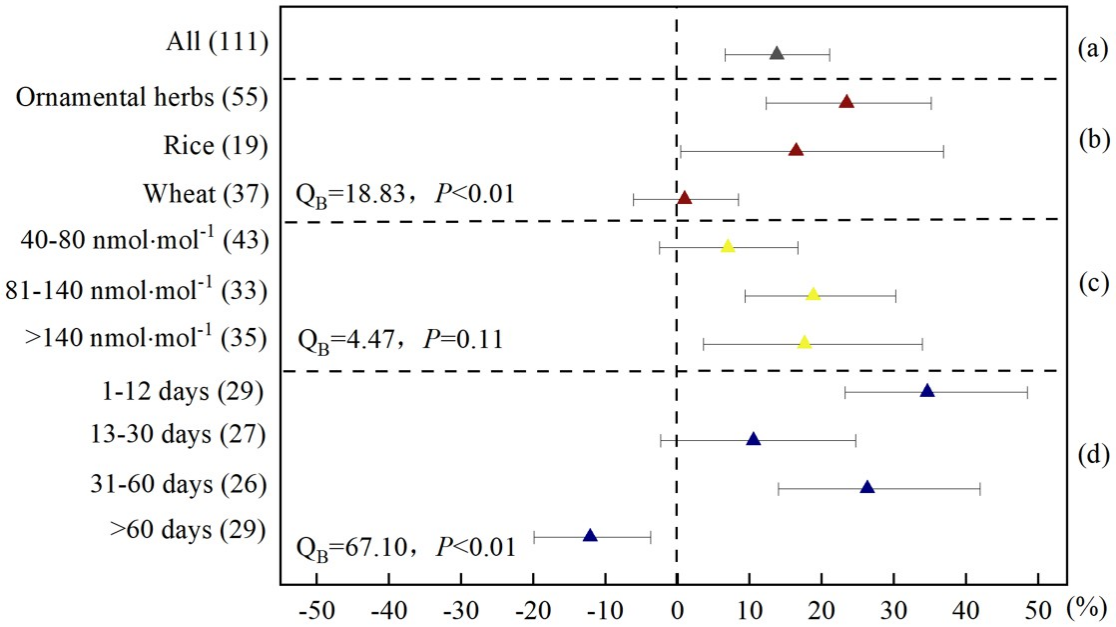
Meta-analysis of the effects of elevated O_3_ on SOD activity of herbaceous plants. (a) categorized into (b) plant species, (c) O_3_ concentration, and (d) experiment duration. SOD activity responses were expressed as the relative increase (%) compared to the control (CK), with 95% CIs represented by the error bars. The numbers of paired observations are shown in parentheses. Between-group heterogeneity (Q_B_) and between-group probability (*P*) was used to describe the statistical differences in SOD activity responses between the different levels of the categorized factors.

**Fig 4:**
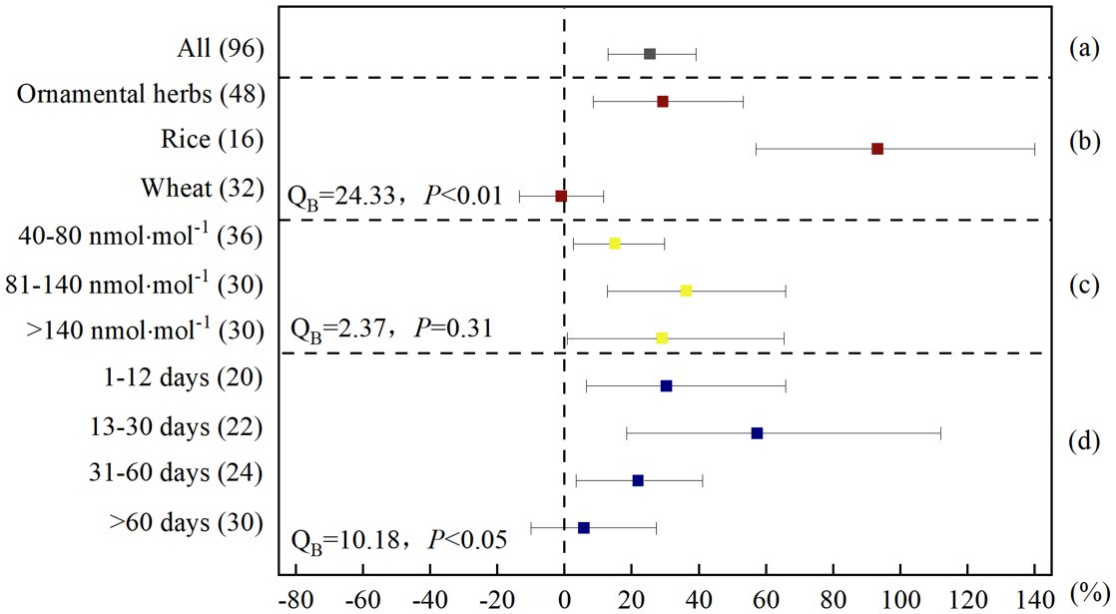
Meta-analysis of the effects of elevated O_3_ on POD activity of herbaceous plants. (a) categorized into (b) plant species, (c) O_3_ concentration, and (d) experiment duration. POD activity responses were expressed as the relative increase (%) compared to the control (CK), with 95% CIs represented by the error bars. The numbers of paired observations are shown in parentheses. Between-group heterogeneity (Q_B_) and between-group probability (*P*) was used to describe the statistical differences in POD activity responses between the different levels of the categorized factors.

Elevated O_3_ significantly increased the activity of antioxidant enzyme across all studies. In this study, SOD and POD activities of herbaceous plants significantly increased by 13.8% (n = 111; Fig 3a) and 25.4% (n = 96; Fig 4a), respectively. SOD and POD are the most important protective enzymes in the plant antioxidant system [45]. In an elevated O_3_ environment, when O_3_ enters the intercellular space of plant leaves, it dissolves in the binding water of the cell wall and forms ROS through a series of chemical reactions that destroy the cell wall and react with unsaturated fatty acids in the cell membrane, causing herbaceous plant leaf damage [46,47]. Antioxidant substances (such as SOD) in plant exosomes react with ROS for primary detoxification and become a defense mechanism against O_3_ damage [48,49]. The remaining ROS that are not eliminated will pass through the plasma membrane and reach the symplast, where the antioxidant components (antioxidant oxidase substances such as POD) in the symplast are induced to participate in redox processes such as the “ascorbate-glutathione (ASA-GSH) cycle” as a secondary detoxification response mechanism, which used to eliminate ROS [45,50,51]. Our results showed that all herbaceous plants in this study mobilized both exosomal and endoplastid antioxidants to defend against elevated O_3_.

When the data were subdivided by plant species, there were significant differences in the response of SOD and POD activities in different plant species to elevated O_3_. The elevated O_3_ increased the SOD activity of ornamental herbs and rice by 23.4% and 16.4%, respectively (Fig 3b); and increased the POD activity of ornamental herbs and rice by 29.3% and 93.1%, respectively (Fig 4b); however, there was no significant effect on SOD and POD activity of wheat (Figs 3b and 4b). Based on the analysis of the activities of these two antioxidant enzymes, the resistance of wheat to high O_3_ concentrations was the weakest, and the resistance of rice was strongest. In our discussion of MDA above, the MDA content of rice increased the least, and we raised the possibility that it may be the least damaged by membrane lipid peroxidation. Here, our results further demonstrated the possibility that this is because antioxidant components, such as POD, in the rice symplast are more resistant to elevated O_3_ than those in ornamental herbs and wheat. From the perspective of antioxidant enzyme activity, the order of O_3_ resistance in the herbaceous plants was rice > ornamental herbs > wheat.

O_3_ concentration were classified in 40–80, 81–140, and > 140 nmol·mol^-1^. Comparing the effects of different O_3_ concentrations on the SOD and POD activities of herbaceous plants, the differences between them were not significant (Figs 3c and 4c). This result is different from some results that have investigated the response of enzyme activity in single plant species to elevated O_3_. We assume that this is because the enzyme activity of herbaceous plants increased significantly when they were exposed to O_3_ concentrations above 40 nmol·mol^-1^. The elevated O_3_ in the atmosphere is increasingly serious and common pollution, and many plants are likely to grow in an environment with an O_3_ concentration of approximately 40–80 nmol·mol^-1^, especially in the summer [41–43], SOD and POD activities in herbaceous plants have been activated. But there is a limit to the enzyme activity in plants: when the concentration of O_3_ in the environment continues to rise, the enzyme activity in plants does not continue to rise.

The different exposure time of herbaceous plants to elevated O_3_ concentrations had a significant effect on SODand POD activity (Figs 3d and 4d). The SOD activity of herbaceous plants increased by 34.6%, 10.5%, and 26.3%, respectively, when exposure time of herbaceous plants in elevated O_3_ environment was 1–12, 13–30 and 31–60 days. When the exposure time was more than 60 days, SOD activity did not increase but significantly decreased by 12.1% (Fig 3d). However, the POD activity of herbaceous plants increased by 30.4%, 57.3%, 21.9% and 5.81%, respectively, when exposure time of herbaceous plants in elevated O_3_ environment was 1–12, 13–30, 31–60 and more than 60 days (Fig 4d). This result may indicate that for most herbaceous plants, when exposed to elevated O_3_ concentration environment for more than 60 days, their antioxidant systems are more severely damaged [52]. At this stage, for most herbs the antioxidant SOD in plant exosomes loses its regulatory ability, and the antioxidant component peroxidase (POD) in the symplast is primarily used for ROS clearance.

### 3.3 Relative importance of explanatory factors

**Fig 5:**
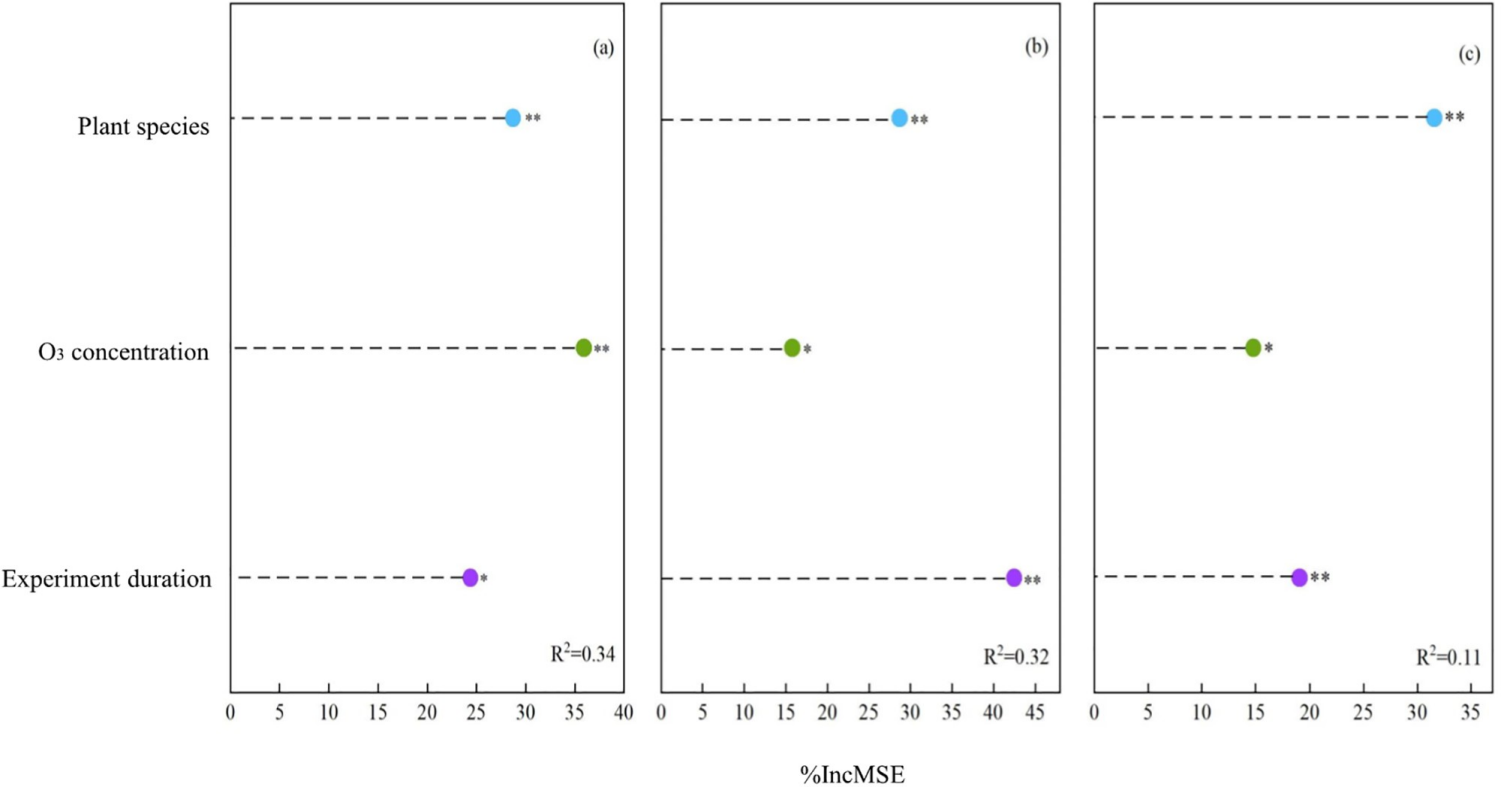
Relative importance (%IncMSE) of explanatory factors in oxidation and antioxidant indexes responses to elevated O_3_. (a) MDA, (b) SOD, and (c) POD. *P*-values < 0.05 are considered significant. * *P* < 0.05; ** *P* < 0.01.

The %IncMSE results of random forest algorithm represent the relative importance of factors with significant influence on elevated O_3_ -induced changes in oxidation and antioxidant indexes (Fig 5). The environmental O_3_ concentration was identified as the most important factor to induced the increased of MDA content, followed by plant species and duration of O_3_ stress in plants (Fig 5a). The results may suggest that when the concentration of O_3_ in the environment exceeds 40 nmol·mol^-1^, it is likely to cause membrane lipid peroxidation damage to plants, even if some damage is not significantly observed. The important factors affected the activity of SOD and POD in plants were not consistent (Fig 5b and 5c), which may be a new discovery. The duration of O_3_ stress in plants was more important than plant species and O_3_ concentration for SOD activity. However, for POD activity, plant species was the most important factor. There were probably two reasons for this. First, there are significant differences in the response of POD activity of different plants to elevated O_3_ environment. The second is that rice and wheat may be more affected by human factors, such as fertilization, than ornamental herbs. At the same time, the result of POD activity R^2^ = 0.11 (Fig 5c), which was lower than that of MDA and SOD (Fig 5a and 5b), which may also indicate that there are other important factors affecting POD activity. In the future, to study the effect of elevated O_3_ on POD activity, human factors (such as fertilization, pesticide spraying, etc.) may need to be included in meta-analysis to further verify this.

## 4. Conclusions

A comprehensive understanding of the effects of elevated O_3_ levels on the antioxidant systems of herbaceous plants is important for exploring effective strategies to mitigate the harmful effects of O_3_ pollution on plants. The present meta-analysis revealed that (1) the antioxidant enzyme activities of ornamental herbs, rice, and wheat were not similar in response to elevated O_3_. Rice is more resistant to elevated O_3_ than wheat and ornamental herbs likely because of the higher activity of antioxidant components (e.g. POD) in the symplasts; (2) exposure to elevated O_3_ concentrations more than60 days, may result in antioxidant SOD lose its regulatory ability, and the antioxidant component POD in the symplast is mainly used to resist O_3_ damage, and (3) the important factors affected the activity of SOD and POD in plants were not consistent: the duration of O_3_ stress in plants was more important than plant species and O_3_ concentration for SOD activity. However, for POD activity, plant species was the most important factor. The limited number of literature studies available for our meta-analysis indicates that O_3_ stress on herbaceous plants from an antioxidant enzyme activity perspective remains underexplored and reported.

## Supporting information

S1 File PRISMA checklist, S1 Fig, and Appendix. (DOCX)

S2 File Data for meta-analysis in this study. (XLSX)
