# Supplementary material for: A meta-analysis of elevated O3 effects on herbaceous plants antioxidant oxidase activity
Source: PLoS One. 2024 Jun 25;19(6):e0305688. doi: 10.1371/journal.pone.0305688 (PMC11198797; doi:10.1371/journal.pone.0305688)
Supplement: S1 File — (DOCX) [file pone.0305688.s001.docx]

**SUPPORTING INFORMATION**

**A meta-analysis of** **elevated O_3_ effects on herbaceous plants antioxidant oxidase activity**

Yi Zhao^1,2^*, Bing Guo^1^, Zhouli Liu^3,4*^, Xiaohan Wang^1^, Guangmin Xiao^5^, Roland Bol^2,6^*

^1^ School of Chemistry and Environmental Engineering, Liaoning University of Technology, Jinzhou, Liaoning 121001, China

^2^ Institute of Bio- and Geosciences, Agrosphere (IBG-3), Forschungszentrum Jülich GmbH, 52425 Jülich, Germany

^3^ College of Life Science and Engineering, Shenyang University, Shenyang 110044, China

^4^ Key Laboratory of Black Soil Evolution and Ecological Effect, Ministry of Natural Resources, Shenyang 110000, China

^5^ Institute of Agricultural Resources and Environment, Hebei Academy of Agriculture and Forestry Sci-ence/Hebei Fertilizer Technology Innovation Center, Shijiazhuang 050051, China

^6^ School of Natural Sciences, Environment Centre Wales, Bangor University, Bangor, LL572UW, U.K.

*** Corresponding author

E-mail: [zhao_yi_66@163.com](mailto:zhao_yi_66@163.com) (YZ); [r.bol@fz-juelich.de](mailto:r.bol@fz-juelich.de) (RB); [zlliu@syu.edu.cn](mailto:zlliu@syu.edu.cn) (ZL)

The Supporting Information contains:

Number of pages:8

Figure:1

**PRISMA Checklist**

| **Section and Topic** | **Item #** | **Checklist item** | **Reported on page** |
| --- | --- | --- | --- |
| **TITLE** | | |  |
| Title | 1 | Identify the report as a systematic review. | 1 |
| **ABSTRACT** | | |  |
| Abstract | 2 | See the PRISMA 2020 for Abstracts checklist. | 2 |
| **INTRODUCTION** | | |  |
| Rationale | 3 | Describe the rationale for the review in the context of existing knowledge. | 3, 4 |
| Objectives | 4 | Provide an explicit statement of the objective(s) or question(s) the review addresses. | 4, 5 |
| **METHODS** | | |  |
| Eligibility criteria | 5 | Specify the inclusion and exclusion criteria for the review and how studies were grouped for the syntheses. | 5;  Supporting Information Fig. S1 |
| Information sources | 6 | Specify all databases, registers, websites, organisations, reference lists and other sources searched or consulted to identify studies. Specify the date when each source was last searched or consulted. | 5 |
| Search strategy | 7 | Present the full search strategies for all databases, registers and websites, including any filters and limits used. | 5 |
| Selection process | 8 | Specify the methods used to decide whether a study met the inclusion criteria of the review, including how many reviewers screened each record and each report retrieved, whether they worked independently, and if applicable, details of automation tools used in the process. | 5;  Supporting Information Fig. S1 |
| Data collection process | 9 | Specify the methods used to collect data from reports, including how many reviewers collected data from each report, whether they worked independently, any processes for obtaining or confirming data from study investigators, and if applicable, details of automation tools used in the process. | 5, 6 |
| Data items | 10a | List and define all outcomes for which data were sought. Specify whether all results that were compatible with each outcome domain in each study were sought (e.g. for all measures, time points, analyses), and if not, the methods used to decide which results to collect. | 5, 6 |
|  | 10b | List and define all other variables for which data were sought (e.g. participant and intervention characteristics, funding sources). Describe any assumptions made about any missing or unclear information. | N/A |
| Study risk of bias assessment | 11 | Specify the methods used to assess risk of bias in the included studies, including details of the tool(s) used, how many reviewers assessed each study and whether they worked independently, and if applicable, details of automation tools used in the process. | 7 |
| Effect measures | 12 | Specify for each outcome the effect measure(s) (e.g. risk ratio, mean difference) used in the synthesis or presentation of results. | 6, 7 |
| Synthesis methods | 13a | Describe the processes used to decide which studies were eligible for each synthesis (e.g. tabulating the study intervention characteristics and comparing against the planned groups for each synthesis (item #5)). | 5, 6 |
|  | 13b | Describe any methods required to prepare the data for presentation or synthesis, such as handling of missing summary statistics, or data conversions. | 5, 6 |
|  | 13c | Describe any methods used to tabulate or visually display results of individual studies and syntheses. | 7 |
|  | 13d | Describe any methods used to synthesize results and provide a rationale for the choice(s). If meta-analysis was performed, describe the model(s), method(s) to identify the presence and extent of statistical heterogeneity, and software package(s) used. | 6, 7 |
|  | 13e | Describe any methods used to explore possible causes of heterogeneity among study results (e.g. subgroup analysis, meta-regression). | 6, 7 |
|  | 13f | Describe any sensitivity analyses conducted to assess robustness of the synthesized results. | 7 |
| Reporting bias assessment | 14 | Describe any methods used to assess risk of bias due to missing results in a synthesis (arising from reporting biases). | 7 |
| Certainty assessment | 15 | Describe any methods used to assess certainty (or confidence) in the body of evidence for an outcome. | 6, 7 |
| **RESULTS** | | |  |
| Study selection | 16a | Describe the results of the search and selection process, from the number of records identified in the search to the number of studies included in the review, ideally using a flow diagram. | 5;  Supporting Information Fig. S1;  PRISMA_diagram |
|  | 16b | Cite studies that might appear to meet the inclusion criteria, but which were excluded, and explain why they were excluded. | N/A |
| Study characteristics | 17 | Cite each included study and present its characteristics. | The list of 31 reports from which the data were extracted for this meta-analysis in the supporting information. |
| Risk of bias in studies | 18 | Present assessments of risk of bias for each included study. | 7 |
| Results of individual studies | 19 | For all outcomes, present, for each study: (a) summary statistics for each group (where appropriate) and (b) an effect estimate and its precision (e.g. confidence/credible interval), ideally using structured tables or plots. | 8, 9,10,11 |
| Results of syntheses | 20a | For each synthesis, briefly summarise the characteristics and risk of bias among contributing studies. | 12 |
|  | 20b | Present results of all statistical syntheses conducted. If meta-analysis was done, present for each the summary estimate and its precision (e.g. confidence/credible interval) and measures of statistical heterogeneity. If comparing groups, describe the direction of the effect. | 8, 9,10,11 |
|  | 20c | Present results of all investigations of possible causes of heterogeneity among study results. | 8, 9,10,11 |
|  | 20d | Present results of all sensitivity analyses conducted to assess the robustness of the synthesized results. | 7,8, 9,10 |
| Reporting biases | 21 | Present assessments of risk of bias due to missing results (arising from reporting biases) for each synthesis assessed. | N/A |
| Certainty of evidence | 22 | Present assessments of certainty (or confidence) in the body of evidence for each outcome assessed. | 7,8, 9,10 |
| **DISCUSSION** | | |  |
| Discussion | 23a | Provide a general interpretation of the results in the context of other evidence. | 8, 9,10,11 |
|  | 23b | Discuss any limitations of the evidence included in the review. | 11 |
|  | 23c | Discuss any limitations of the review processes used. | N/A |
|  | 23d | Discuss implications of the results for practice, policy, and future research. | 11 |
| **OTHER INFORMATION** | | |  |
| Registration and protocol | 24a | Provide registration information for the review, including register name and registration number, or state that the review was not registered. | The protocol is described in the Methods. Registration does not apply |
|  | 24b | Indicate where the review protocol can be accessed, or state that a protocol was not prepared. | N/A |
|  | 24c | Describe and explain any amendments to information provided at registration or in the protocol. | N/A |
| Support | 25 | Describe sources of financial or non-financial support for the review, and the role of the funders or sponsors in the review. | 13 |
| Competing interests | 26 | Declare any competing interests of review authors. | 13,14 |
| Availability of data, code and other materials | 27 | Report which of the following are publicly available and where they can be found: template data collection forms; data extracted from included studies; data used for all analyses; analytic code; any other materials used in the review. | When the manuscript is accepted, we will make it available upon editor's request |

**
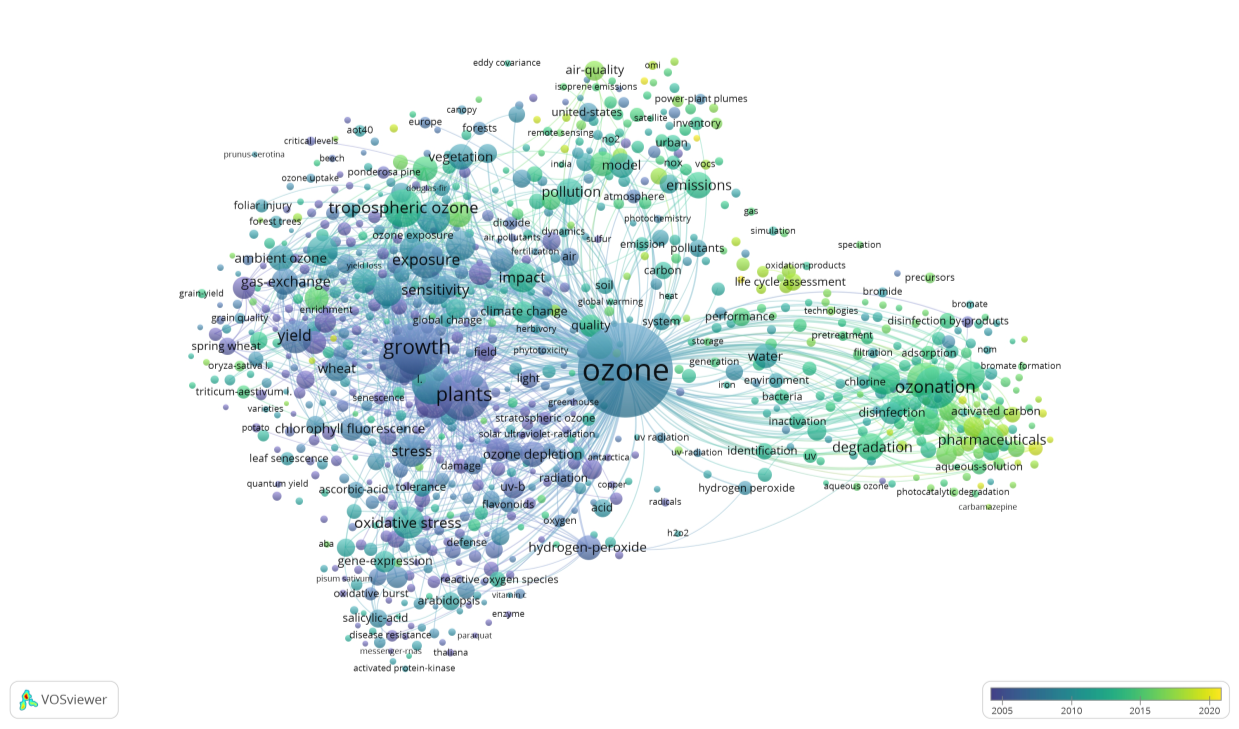
**

**Fig S1. Keyword network cluster graph in English research reports**

**Appendix. The list of 31 papers from which the data were extracted for this meta-analysis.**

1. Zhuang, M.H.; Li, Y.C.; Li, Y.; Guo, Z.W.; Yang, Q.P.; Gu, D.X.; Chen, S.L. Physiological responses of three dwarf ornamental bamboos to the elevated atmospheric ozone concentration (in Chinese). Acta Botanica Boreali-Occidentalia Sinica, 2011, 31(10), 2014–2020.

2. Feng, Z.Z.; Pang, J.; Kobayashi, K.; Zhu, J.G.; Ort, D.R. Differential responses in two varieties of winter wheat to elevated ozone concentration under fully open-air field conditions. Global Change Biology, 2011, 17, 580–591.

3. Feng, Z.Z.; Wang, L.; Pleijel, H.; Zhu, J.G.; Kobayashi, K. Differential effects of ozone on photosynthesis of winter wheat among cultivars depend on antioxidative enzymes rather than stomatal conductance. Science of the Total Environment, 2016, 572, 404–411.

4. Scebba, F.; Soldatini, G.; Ranieri, A. Ozone differentially affects physiological and biochemical responses of two clover species; Trifolium repens and Trifolium pratense. Environmental Pollution, 2003, 123, 209–216.

5. Wang, J.L.; Zeng, Q.; Zhu, J.G.; Chen, C.; Liu, G.; Tang, H.Y. Apoplastic antioxidant enzyme responses to chronic free-air ozone exposure in two different ozone-sensitive wheat cultivars. Plant Physiology and Biochemistry, 2014, 82, 183–193.

6. Yang, N.; Wang, X.K.; Zhang, Y.L.; Zheng, F.X.; Chen, Y.Y. Ozone injury to marigold (Tagetes erecta Linn.), petunia (Petunia hybridda Vilm.) and morning glory (Pharbitis purpurea (L.) Voigt) and their different physiologica responses(in Chinese). Asian Journal of Ecotoxicology, 2017, 12(6), 141–149.

7. Jin, M.H.; Feng, Z.W. Effects of ozone on membrane protective system of winter wheat leaves (in Chinese). Acta Ecologica Sinica, 2000, 20(3), 444–447.

8. Zhang, W.W.; Zheng, F.X.; Wang, X.K.; Feng, Z.Z.; Ouyang, Z.Y. Effects of ozone on root activity, soluble protein content and antioxidant system in Oryza Sativa roots (in Chinese). Chinese Journal of Plant Ecology, 2009, 33(3), 425–432.

9. Jin, M.H.; Feng, Z.W.; Zhang, F.Z. Effects of ozone on membrane lipid peroxidation and antioxidant system of rice leaves (in Chinese). Environmental Science, 2000, 21(3), 1–5.

10. Zheng, Q.W.; Wang, X.K.; Feng, Z.Z.; Song, W.Z.; Feng, Z.W. Ozone effects on chlorophyll content and lipid peroxidation in the in situ leaves of winter wheat (in Chinese). Acta Botanica Boreali-Occidentalia Sinica, 2005, 25(11), 2240–2244.

11. Qin, Z.Q.; Xu, S.; Qi, S.Y.; Chen, W.; He, X.Y.; Wang, Y.J. Effects of elevated O3 concentration and warming on oxidative jury and antioxidant enzyme activities in leaves of Cleome spinosa (in Chinese). Chinese Journal of Ecology, 2020, 39(3), 830–837.

12. Xiong, D.L.; Yu, X.; Xu, S.; Deng, L.L.; He, X.Y.; Chen, W. Effects of exogenous abscisic acid on petunias under ozone stress (in Chinese). Northern Horticulture, 2018, 4, 96–102.

13. Zhang, W.W.; Zheng, F.X.; Wang, X.K.; Feng, Z.Z.; Ouyang, Z.Z.; Feng, Z.W. Effects of elevated ozone on rice (oryza sativa L.), leaf lipid peroxidation and antioxidant syste (in Chinese). Chinese Journal of Applied Ecology, 2008, 19(11), 2485–2489.

14. Zheng, Y.F.; Zhang, J.E.; Wu, R.J.; Zhao, Z.; Hu, C.D. Effects of ozone stress on photosynthesis and physiological characteristics of winter wheat in northern china (in Chinese). Journal of Agro-Environment Science, 2010, 29(8), 1429–1436.

15. Xu, L.; Zhao, T.H.; Hu, Y.Y.; Shi, Y. Effects of high concentration ozone on membrane lipid peroxidation and antioxidant system of spring wheat (in Chinese). Agricultural Research in the Arid Areas, 2008, 26(2), 74–78.

16. Wang, Y.J.; Li, Y.; Xu, S.; He, X.Y.; Chen, W.; Wu, X. Effects of elevated ozone concentrations on photosynthetic and resistant physiological characteristics of Monarda didyma L. leaves (in Chinese). Chinese Journal of Ecology, 2019, 38(3), 696–703.

17. Yang, J.L.; Xu, S.; Ma, C.L.; Han, Z.S.; Li, D.; Li, Y.; Wang, N.; Wang, Y.J.; He X.Y. Comparison of physiological characteristics of two ornamental grass species under elevated ozone concentrations (in Chinese). Acta Ecologica Sinica, 2021, 41(19), 7763–7773.

18. Zhao, Y.; Xu, S.; He, X.Y.; Chen, W. Physiological response of two Lolium varieties to the enhancements of tropospheric O3 concentration (in Chinese). Acta Agrestia Sinica, 2015, 23(5), 1013–1020.

19. Zhuang, M.H.; Li, Y.C.; Chen, S.L. Differences in O3 stress tolerance between Phyllostachys edulis and Oligostachyum lubricum (in Chinese). Chinese Journal of Ecology, 2011, 30(10), 2191–2196.

20. Ping, Q.; Xu, S.; Li, J.; He, X.Y.; Chen, W.; Huang, Y.Q. Ecophysiological responses of turf-type white clover (Trifolium repens) to elevated O3 concentration(in Chinese). Chinese Journal of Ecology, 2017, 36(5), 1234–1242.

21. Zhao, Y.; Xu, S.; He, X.Y.; Chen, W.; Li, M.; Zhang, N.; Fu, W. Physiological responses of three cool-season types of turfgrass to elevated O3 concentrations (in Chinese). Chinese Journal of Ecology, 2014, 33(12), 3203–3208.

22. Yang, J.L. Physiological response of three ornamental grass species to atmospheric increasing temperature and ozone concentration (in Chinese). Southwest Forestry University, 2020, Yunnan.

23. Wu, F.F.; Zheng, Y.F.; Wu, R.J.; Wang, J.Q. Concentration of O_3_ at the atmospheric surface affects the changes characters of antioxidant enzyme activities in Triticum aestivum (in Chinese). Acta Ecologica Sinica. 2011, 31(4), 4019-4026.

24. Zheng, Q.W.; Wang, X.K.; Xie, J.Q.; Feng, Z.Z.; Feng, Z.W.; Ni, X.W.; Ouyang, Z.Y. Effects of exogenous ascorbate acid on membrane protective system of in situ rice leaves under O_3_ stress (in Chinese). Acta Ecologica Sinica, 2006, 26(4), 1131-1137.

25. Liu, X.; Sui, L.H.; Huang, Y.Z.; Geng, C.M.; Yin, B.H. Physiological and visible injury responses in different growth stages of winter wheat to ozone stress and the protection of spermidine. Atmospheric Pollution Research, 2015, 6, 596–604.

26. Xu, S.; Li, Y.; Li, B.; He, X.Y.; Chen, W.; Yan, K. Responses of growth, oxidative injury and chloroplast ultrastructure in leaves of Lolium perenne and Festuca arundinacea to elevated O_3_ concentrations. International Journal of Molecolar Sciences, 2022, 23(9), 5153–5168.

27. Zhang, X.X.; Zhang, X.F.; Zhang, L.; Zhang, Y.C.; Zhang, D.; Gu, X.; Zheng, Y.H.; Wang, T.Z.; Li, C.H. Metabolite profiling for model cultivars of wheat and rice under ozone pollution. Environmental and experimental botany, 2020, 179, 104214.

28. Su, B.Y.; Zhou, M.H.; Xu, H.; Zhang, X.J.; Li, Y.G.; Su, H.; Xiang, B. Photosynthesis and biochemical responses to elevated O3 in Plantago major and Sonchus oleraceus growing in a lowland habitat of northern China. Journal of Environmental Sciences, 2017, 53, 113–121.

29. Ueda, Y.; Uehara, N.; Sasaki, H.; Kobayashi, K. Impacts of acute ozone stress on superoxide dismutase (SOD) expression and reactive oxygen species (ROS) formation in rice leaves. Plant Physiology and Biochemistry, 2013, 70, 396–402.

30. Wang, J.L.; Liu, G.; Liu, F.X.; Zhou, J.G. Responses of antioxidant enzymes to chronic free air ozone stress in rice (Oryza sativa L.) cultivars with diferent ozone sensitivities. Bulletin of Environmental Contamination and Toxicology, 2019, 103(3), 428–434.

31. Wu, X. Physiological responses of two Zoysia species to elevated O_3_ concentrations (in Chinese). Anhui Agricultural University, 2018, Anhui.
